# Prognostic value of stem cell markers in esophageal and esophagogastric junction cancer: a meta-analysis

**DOI:** 10.7150/jca.33699

**Published:** 2020-04-27

**Authors:** Elisabetta Trevellin, Giovanni Pirozzolo, Matteo Fassan, Roberto Vettor

**Affiliations:** 1 Department of Medicine, Endocrine-Metabolic Laboratory, University of Padua, 35128 Padua, Italy; 2 Emergency General Surgery, Ospedale dell'Angelo - ULSS 3 Serenissima, 30174 Venice, Italy; 3 Department of Medicine, Surgical Pathology Unit, University of Padua, 35128 Padua, Italy

**Keywords:** Esophageal cancer, Cancer stem cells, CD34, CD133, Nucleostemin, Neoadjuvant therapy, Prognosis

## Abstract

**Background**: Esophageal cancer is an aggressive tumor, with poor prognosis and low survival rates. Although diagnosis and treatment have improved considerably, more efficient prognostic factors are urgently needed to prevent postoperative recurrence and metastasis. Cancer stem cells are key players in tumor progression and several studies have investigated the association between the expression of stemness genes and clinical outcome. However, the prognostic value of stemness markers in esophageal cancer remains controversial. We identified six factors involved in angiogenesis, anti-apoptosis and self-renewal that have been associated to poor prognosis in other types of cancer. We conducted a review of the literature and a meta-analysis to assess their potential prognostic role in this malignancy.

**Material and Methods**: The database of PMC, PubMed, Web of Science, Embase and The Cochrane Library were searched to investigate the association between CD34, CD133, Nucleostemin, OCT-4, NANOG and CD90, and the survival of patients affected by esophageal squamous cell carcinoma or esophageal adenocarcinoma. Among the 615 eligible studies, a total of 19 articles (including 1586 patients) met the inclusion criteria for the meta-analysis, and the pooled hazard ratio and 95% confidence intervals were calculated.

**Results**: Data showed that high expression of CD34 (HR 2.10; 95%CI 1.41-3.14; I^2^=56%; p=0.0003), CD133 (HR 1.91; 95%CI 1.15-3.19; I^2^=55%; p=0.01) and Nucleostemin (HR 2.97; 95%CI 1.11-7.98; I^2^=0%; p=0.03) were associated with poor prognosis in patients affected by esophageal cancer. The expression of NANOG and OCT-4 showed no significant association with survival of patients, whereas no study involving CD90 was included in this meta-analysis. **Conclusion:** CD34, CD133 and Nucleostemin might represent useful prognostic markers in patients affected by esophageal cancer.

## Introduction

Esophageal cancer is one of the most unknown and deadliest cancers worldwide, characterized by an aggressive nature and poor survival rate 1. Esophageal cancer typically occurs in two histologic forms: squamous cell carcinoma (SCC) is the predominant form, arising from the stratified squamous epithelial lining of the organ, whereas esophageal adenocarcinoma (EAC) affects columnar glandular cells that replace the squamous epithelium 2. Although current treatment options (including surgery, radiation and chemotherapy) are constantly improving, the overall survival remains poor and stronger prognostic factors are needed. Cancer stem cells (CSCs) may play an important role in the progression and prognosis of esophageal cancers, by expressing factors involved in angiogenesis, neoangiogenesis, anti-apoptosis and self-renewal 3. Some studies showed that a higher expression of the stemness markers CD34, CD90, Nucleostemin, CD133, OCT-4 and NANOG in tumoral tissue of patients correlated with poor prognosis in different types of tumors. In particular, CD34 is reduced in breast cancer 4 and prostate cancer 5 after neoadjuvant treatment and is usually related to VEGF expression, which negatively correlates to the response to neoadjuvant therapy in esophageal cancer tissue 6. CD133 has been shown to be associated with worse prognosis in NSCLC 7, ovarian cancer 8, hepatocellular carcinoma 9, breast cancer 10 and colorectal cancer 11, however its role in esophageal cancer still needs to be investigated. CD90 is upregulated in cancer-associated fibroblasts and correlated with recurrence in hepatocellular carcinoma 12 and with survival in neuroblastoma 13*.* Nucleostemin is upregulated in recurrent esophageal carcinoma 14, in advanced malignant phenotype of oral squamous cell carcinoma 15 and in human breast cancer cells resistant to chemotherapy 16. OCT-4 is essential for anti-apoptosis in chemoresistant cell lines 17 and is increased in tumor treated with neoadjuvant therapy 18. NANOG is an early-differentiation marker that has been associated with worse prognosis in tongue squamous cell carcinoma 19, ovarian serous carcinoma 20 and breast cancer 21. The analysis of these markers in esophageal cancer tissue may potentially lead to better prognosis as well as play a part in assessing tumor response to therapy; however, their prognostic role is still not clear to date.

Antibody-based agents like ipilimumab, pembrolizumab, and nivolumab are currently used to block CTLA-4 and PD-1 binding to PDL-1 respectively, effectively enhancing endogenous immune responses and antitumor activity. Esophageal cancer is also being explored in terms of immune checkpoint inhibition trials and early results seem promising in esophageal SCC and gastric adenocarcinoma 22. Recent studies implicated CSCs to play a role in tumor chemoradio-resistance and response to CRT so CSC markers might be used to select patients who would not benefit from conventional CRT but would need other therapy such as immunotherapy 23. On the other hand, as observed in glioma, CSCs (CD133-positive cells) are able to repair DNA damage more efficiently and rapidly than CD133 negative cells and this might decrease the tumor mutational load and, by consequence, tumor immunogenicity 24. Therefore, the use of CSC markers to predict the need and the effect of immunotherapy is still under debate. In this study, we collected the data available in literature and conducted a meta-analysis to clarify the prognostic value for each marker in esophageal cancer.

## Materials and Methods

### Literature search and eligibility Criteria

This review was registered with the International Prospective Register for Systematic Reviews (PROSPERO) platform under the number: CRD42017058771. The database of PMC, PubMed, Web of Science, Embase and The Cochrane Library were searched in March 2017 and the search strategy was the following: (CD34 OR CD90 OR Nucleostemin OR CD133 OR "OCT4" OR "OCT-4" OR NANOG) AND (esophageal OR oesophageal OR esophagus OR oesophagus OR esophagogastric junction) AND (cancer OR tumor OR carcinoma OR adenocarcinoma OR neoplasm). The inclusion criteria were: 1) the diagnosis of SCC or EAC was based on pathological examination; 2) the expression of CD34 or CD90 or Nucleostemin or CD133 or OCT-4 or NANOG with OS/DSS/DFS was reported; 3) HRs and 95% CIs were provided in text or sufficient data was provided for the calculation of HRs and 95% CIs; 4) articles published as original research. To avoid duplicate data, we selected only the more recent or complete article when multiple reports described the same population. The exclusion criteria were: 1) reviews, meeting abstracts, letters; 2) non-primitive tumors; 3) case-reports; 4) animal or *in vitro* studies; 5) sample size <10 patients. Two researchers (E.T. and M.S.) independently selected studies that matched the inclusion criteria. Any discordance was resolved by discussion.

### Data extraction

Two researchers (E.T. and M.S.) independently extracted the following data: author, year of publication, study center and country, sample size, demographic data, clinicopathological parameters, cut-off value of CD34 or CD90 or Nucleostemin or CD133 or OCT-4 or NANOG expression, survival data, follow up duration, tumor location, neoadjuvant therapy characteristics, methodological data, overall survival (OS) hazard ratio (HR), disease free survival (DFS) HR, progression free survival (PFS) HR. HR were extracted both from multivariate and univariate analysis, preferring data from multivariate analysis when available. When HR was not declared it was extracted from Kaplan-Meyer curves following the method described by Parmar 25.

### Quality assessment

Two researchers (E.T. and M.S.) independently assessed the quality of included studies by the Newcastle-Ottawa Scale (NOS) for assessing the quality of non-randomised studies in meta-analyses. Studies with NOS scores of less than 5 were not included in the meta-analysis.

### Statistical analysis

Extracted data were analysed using RevMan 5.3 analysis software. Generic inverse variance was used to pool hazard ratios. Fixed-effect model and random-effect model were used depending on heterogeneity. Heterogeneity, assessed using I^2^ statistic, was considered relevant when >30% 26. Funnel plot asymmetry was estimated by visual inspection to assess publication bias of the included studies for each stemness marker 26. Statistical significance was considered relevant when p<0.05.

## Results

### Study Characteristics

**Figure 1:**
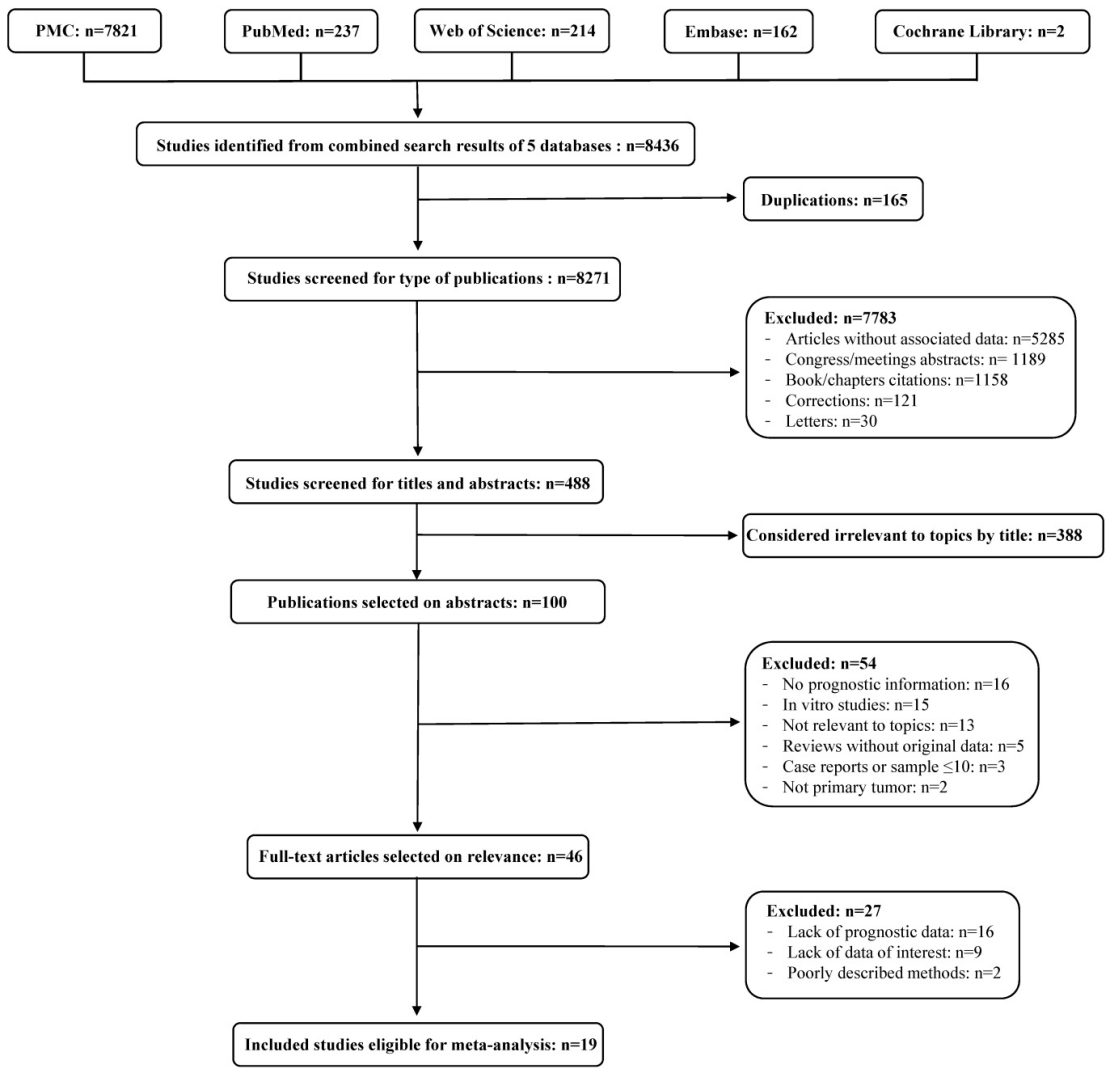
** Flow diagram of the literature review process.** Identified, included and excluded studies are shown in the different steps of the inspection.

The selection process of the eligible studies is presented in Figure 1. A total of 19 articles 14, 27-44 including 1586 patients met the inclusion criteria for the meta-analysis. The basic characteristics of each eligible study and the NOS scores of each study are summarized in Table 1. All articles were published between 1996 and 2017, most of the studies were conducted in Asia (n=15) and the remaining were conducted in Europe (n=4). Seven studies involved the analysis of the marker CD34, five studies the analysis of CD133, four studies the analysis of OCT-4, three studies the analysis of NANOG and two studies the analysis of Nucleostemin, whereas none of the selected studies involved the analysis of CD90. The majority of the studies were conducted on patients affected by SCC (n=18) and, among them, one study 35 also involved patients affected by EAC. One study 27 only involved patients with EAC. The detection method was immunohistochemistry (IHC) for all the studies except for one 30, in which mRNA *in situ* hybridization (ISH) was used. The cut-off values were expressed as percentage of positive cells, number of positive cells or as a score determined on the intensity of immunohistochemical expression. Eleven studies explored the prognostic value of a marker in overall survival (OS), three studies in cumulative survival (CS), two studies in disease-free survival (DFS), two studies in the onset of lymphatic metastasis, one study in relapse-free survival (RFS), one study in cause-specific survival (CSS) and one in esophageal cancer-specific survival (ECSS). The information regarding name (clones), manufacturer and (if available) dilution of the antibodies used to perform the staining in each study are summarized in Table 2.

### Patient characteristics

The characteristics of patients involved in the 19 studies included in the meta-analysis are summarized in Table 3. A total of 1586 patients (1253 male and 360 female) were analyzed, the median age ranged from 42 to 68.2 and the months of follow up ranged from 0.3 to 192. In 17 studies, the stages of tumor (from I to IV) were reported and in 8 studies the location of tumor (lower, middle or upper esophagus) was specified. In 6 studies the patients underwent neoadjuvant therapy (308 patients underwent chemotherapy and 148 underwent radiotherapy) before surgery.

### Prognostic value of CD34 in esophageal cancer

**Figure 2:**
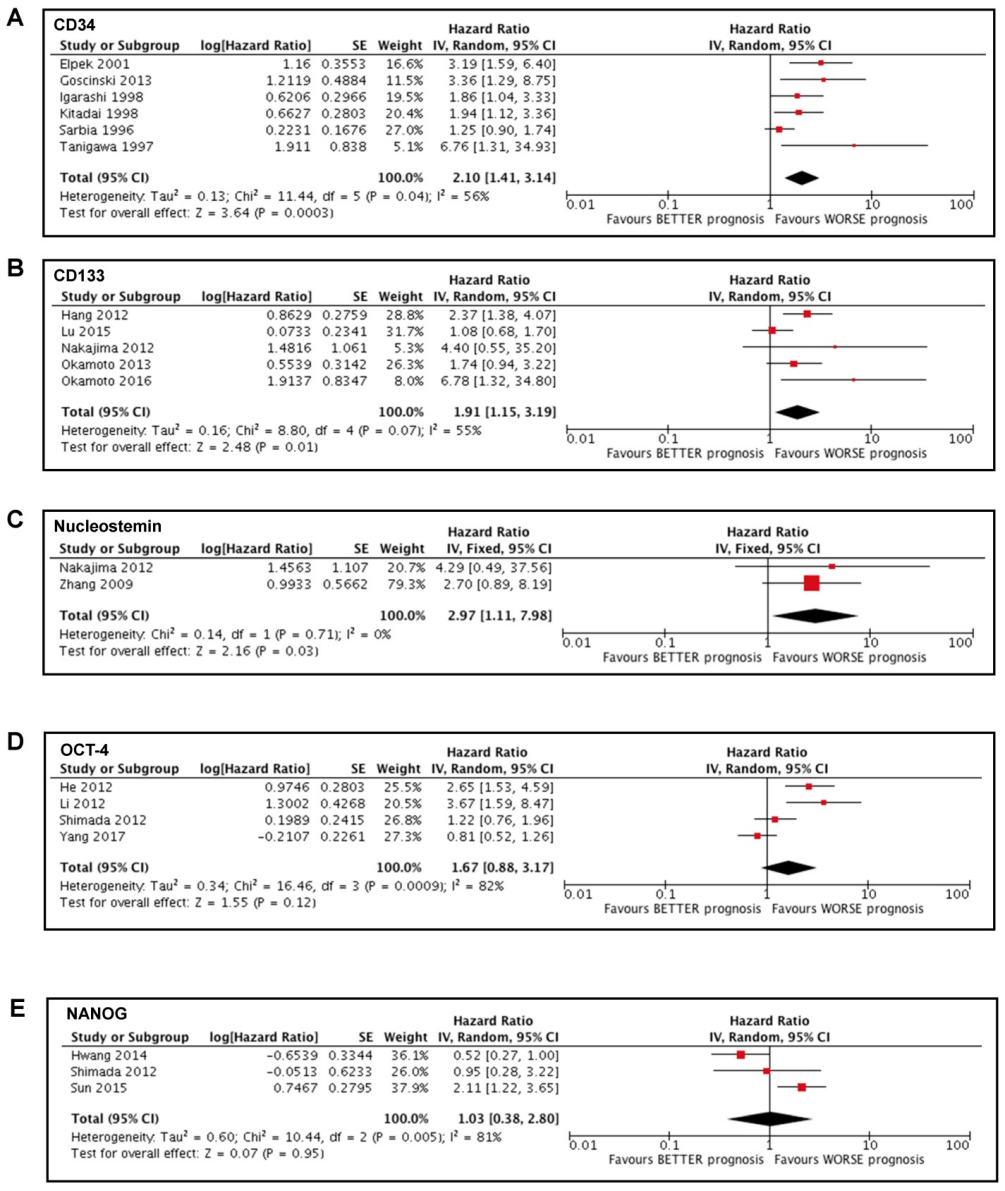
** Forest plots of stemness markers associated with OS.** (A) CD34 overall survival; (B) CD133 overall survival; (C) Nucleostemin overall survival; (D) OCT-4 overall survival; (E) NANOG overall survival.

We conducted a meta-analysis of the studies 35-37, 41, 44 that reported the analysis of the marker CD34. These studies involved 475 patients, most of them with SCC (404) and the remaining 71 with EAC. Tumor was localized mainly in the middle or lower portion of esophagus and most of the patients (396) did not undergo neoadjuvant therapy before surgery (and thus sampling). Due to the presence of only one article involving patients affected by EAC, we decided to exclude those data from the meta-analysis to avoid any bias caused by the different type of tumor. Our study showed a significant association between high CD34 expression in tumoral tissue and a poorer prognosis in patients affected by esophageal cancer (HR 2.10; 95%CI 1.41-3.14; I^2^ 56%; p=0.0003) (Figure 2A).

### Prognostic value of CD133 in esophageal cancer

Studies involving CD133 14, 28, 32, 38, 40 were analyzed. These studies involved 448 patients with SCC. Tumor was localized mainly in the middle portion of esophagus and most of the patients (350) did not undergo neoadjuvant therapy before surgery. The meta-analysis showed a significant association between high expression of CD133 in tumoral tissue and poor prognosis in patients affected by esophageal cancer (HR 1.91; 95%CI 1.15-3.19; I^2^ 55%; p=0.01) (Figure 2B).

### Prognostic value of Nucleostemin in esophageal cancer

Two studies 14, 30 involved the analysis of Nucleostemin and included a total of 113 patients with SCC, of which 51 received neoadjuvant therapy before surgery. The meta-analysis that we carried out showed a significant association between high expression of Nucleostemin in tumoral tissue and poor prognosis in patients affected by esophageal cancer (HR 2.97; 95%CI 1.11-7.98; I^2^ 0%; p=0.03) (Figure 2C).

### Prognostic value of OCT-4 in esophageal cancer

Four studies 29, 31, 33, 34 examined the expression of OCT-4 and involved 411 patients with SCC that did not undergo neoadjuvant therapy before surgery. Our meta-analysis showed no significant association between OCT-4 expression and the survival of patients affected by esophageal cancer (HR 1.67; 95%CI 0.88-3.17; I^2^ 82%; p=0.12) (Figure 2D).

### Prognostic value of NANOG in esophageal cancer

Three studies 29, 39, 42 involved the analysis of NANOG and included a total of 271 patients with SCC, of which 41 underwent preoperative chemoradiation therapy. The meta-analysis that we conducted showed no significant association between the expression of NANOG and the survival of patients affected by esophageal cancer (HR 1.03; 95%CI 0.38-2.80; I^2^ 81%; p=0.95) (Figure 2E).

### Prognostic value of CD90 in esophageal cancer

Among the 19 included studies, we did not find any available data about CD90 expression and prognostic value in patients affected by esophageal cancer; therefore, we were not able to conduct a meta-analysis on this marker.

### Prognostic value of stem cell markers in patients who did not undergo neoadjuvant therapy

**Figure 3:**
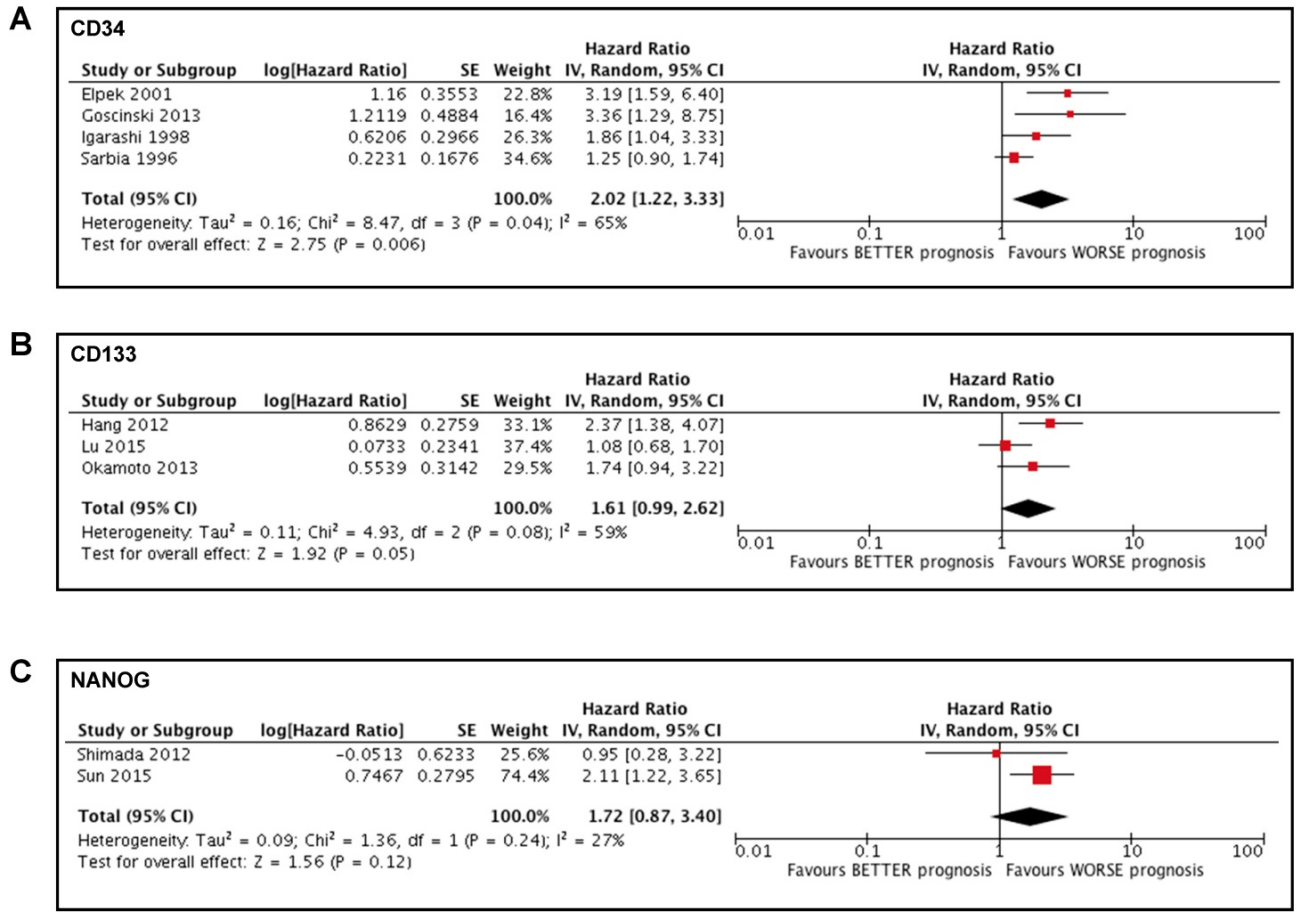
** Forest plots of stemness markers in patients who did not undergo neoadjuvant therapy.** (A) CD34 overall survival; (B) CD133 overall survival; (C) NANOG overall survival.

We conducted a sub-analysis to investigate the potential impact of neoadjuvant treatment on the prognostic value of the stem cells markers, to avoid any bias due to the presence of chemotherapy or radiotherapy. Two studies 41, 43 were excluded from the meta-analysis of CD34 and the sub-analysis showed a significant association between high CD34 expression in tumoral tissue and a poorer prognosis in patients affected by esophageal cancer (HR 2.02; 95%CI 1.22-3.33; I^2^ 65%; p=0.006) (Figure 3A). Two studies^14^, ^32^ were excluded from the meta-analysis of CD133 and the sub-analysis showed a tendentially significant association between high CD133 expression in tumoral tissue and a poorer prognosis in patients affected by esophageal cancer (HR 1.61; 95%CI 0.99-2.62; I^2^ 59%; p=0.05) (Figure 3B). One study 39 was excluded from the meta-analysis of NANOG and the sub-analysis showed no significant association between the expression of NANOG and the survival of patients affected by esophageal cancer (HR 1.72; 95%CI 0.87-3.40; I^2^ 27%; p=0.12) (Figure 3C).

### Publication bias

**Figure 4:**
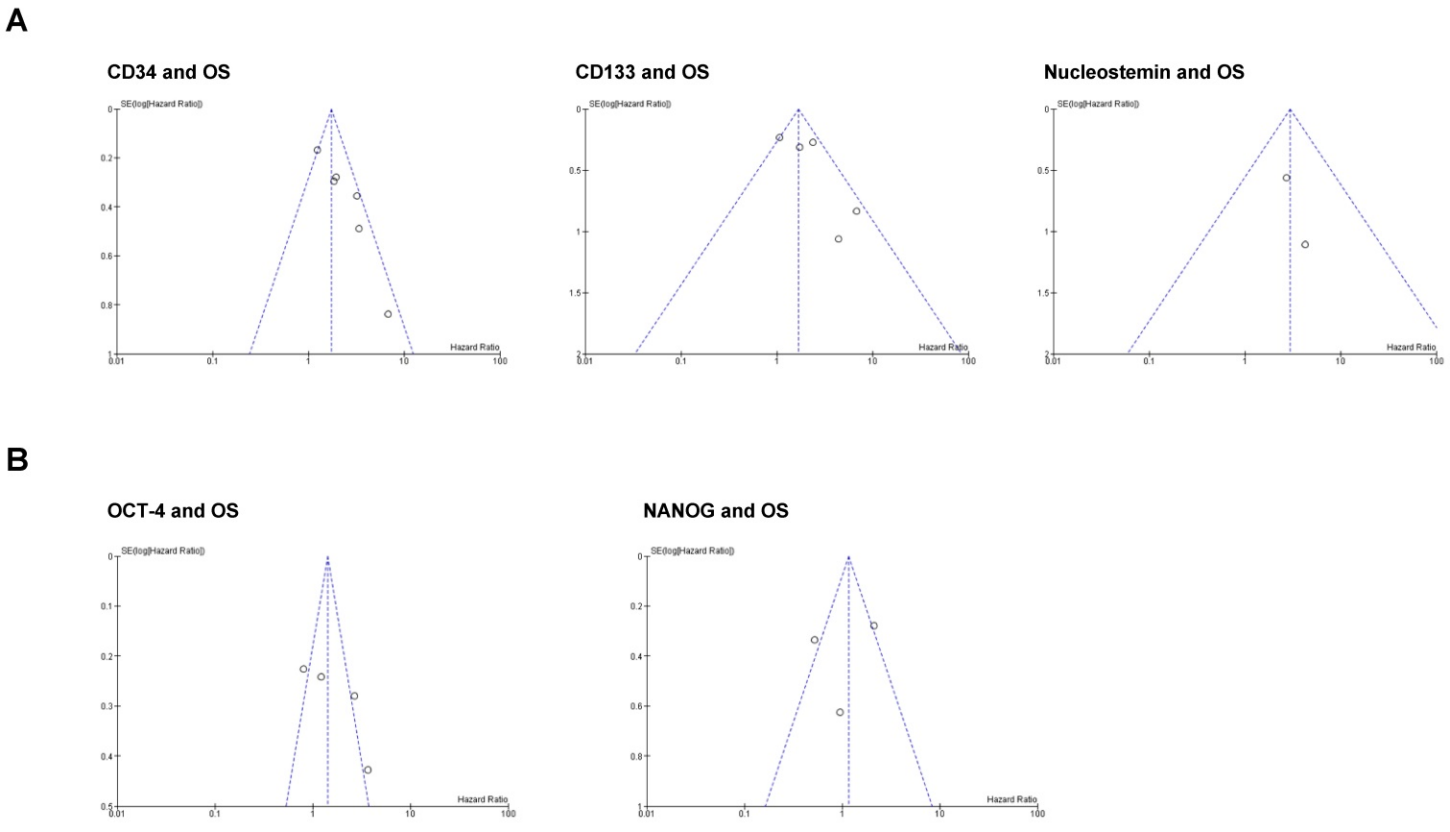
** Funnel plots for publication bias test with 95%confidence limits.** (A) Stemness markers associated with OS; (B) Stemness markers not associated with OS.

We performed funnel plot analysis to assess the presence of publication bias in our meta-analysis (Figure 4). The visual inspection of the plots suggests that asymmetry may be present in the graphs of the studies involving CD34 and CD133, whereas studies involving Nucleostemin, OCT-4 and NANOG seem to be free from publication bias. However, we cannot conclude that a significant publication bias is present because real asymmetry is difficult to distinguish from chance when less than 10 studies are included in the analysis 26.

## Discussion

The emerging role of stemness genes as prognostic markers in esophageal cancer needs to be investigated, in order to identify potential prognostic tools that could be more powerful in the management of these malignancies. We identified data from 19 studies that enrolled 1586 patients with esophageal cancer (SCC or EAC). We conducted the meta-analysis for OS and not for DFS because there were less than two studies reporting DFS for each marker. We found that high expression of CD34, CD133 and Nucleostemin in tumor tissue was associated to a poor prognosis in patients affected by esophageal cancer. Data regarding NANOG and OCT-4 showed no significant association between the expression of these markers and OS of patients, whereas no studies involving the measurement of CD90 expression were included in our meta-analysis.

In the studies involving CD34, this stemness marker was measured mostly to assess microvascular density (MVD) in tumoral tissue, to investigate the potential prognostic value of microvascularization in different analyses. With an HR of 2.10 and a 95%CI from 1.41 to 3.14, CD34 emerges as the strongest prognostic factors among the markers analyzed in this meta-analysis. This result suggests that high MVD has a prognostic value in esophageal cancer, as recently concluded in another meta-analysis 45. Tumor angiogenesis is a multi-step process that allows exchange of nutrients, oxygen and growth factors between cancer cells and blood stream, favoring tumor growth and spread 46. CD34 is expressed by endothelial precursor cells that play a crucial role during angiogenesis, although its function is not fully elucidated 47. High MVD, measured as CD34 expression, has been shown to have significant prognostic value in different types of cancer: it is associated with poor survival in non-small-cell lung carcinoma (NSCLC) 48 and colorectal cancer 49, and with recurrence in bladder 50 and prostate 51 cancer. It is therefore conceivable that the percentage of cells expressing CD34 may represent a powerful tool to also assess prognosis in esophageal cancer patients.

CD133 is a key factor in epithelial-mesenchimal transition processes, it has been recognized as a marker of cancer stem cells in several type of solid tumors 52 and its biological functions include tumor initiation, cellular migration, vasculogenic mimicry and drug resistance 53. Although the role of CD133 has not been fully understood, it has been hypothesized that its expression may confer self-renewal capacity, dedifferentiation/stem cell-like properties and anti-apoptotic behavior to a specific population of esophageal cancer stem cells, thus promoting chemoresistance and tumor recurrence 54. This process may be induced by the inhibition of miR-377, that specifically targets the 3'-UTR binding site of CD133, as suggested by a recent study 55 but the underlying mechanism still needs to be clarified.

Nucleostemin upregulation was associated with poor prognosis in hepatocellular carcinoma 56, breast cancer 57, gastric cancer 58 and oral squamous cell carcinoma 15. In esophageal cells, Nucleostemin may promote cell proliferation via p21 inhibition, as suggested by a study on an esophageal squamous carcinoma cell line 59, but further studies are required to examine more deeply the role of Nucleostemin in tumor progression and/or chemoresistance.

Data involving NANOG and OCT-4 were characterized by elevated heterogeneity (81% and 82% respectively) and we observed a remarkable variability among the cut-off values used in the different studies to determine the positive staining for each marker. In particular, OCT-4 cut-off values ranged from >3 (of a score from 0 to 9) to ≥2 (of a score from 0 to 3), whereas NANOG cut-off ranged from >0 to >8 (of a score from 0 to 12). This may have led to inconclusive results and more studies are therefore needed to assess the prognostic significance of these two markers.

We conducted a sub-analysis to explore the potential effect of neoadjuvant therapy in the prognostic value of the markers that we examined. Given the impossibility of accurately discriminating patients that received neoadjuvant treatment in each study, we decide to exclude the articles involving chemotherapy and/or radiotherapy from the meta-analyses that included them. The sub-analyses showed that, after the exclusion of all the patients that received neoadjuvant treatment, the prognostic value was significant for CD34 while it tended to be significant for CD133 and remained not significant for NANOG. These results are consistent with the results that included patients who underwent neoadjuvant treatment, suggesting that the presence of preoperative chemotherapy or radiotherapy may not affect the prognostic relevance of these markers on the survival of patients affected by esophageal cancer.

This meta-analysis has some limitations that must be taken into consideration: 15 of the 19 included studies were conducted in Asians and the remaining 4 were conducted in Caucasians, which may produce potential population selection bias. Furthermore, not uniform cut-off values to assess high and low expression of stemness markers may have affected the results of this meta-analysis. Moreover, the selected studies that reported data on EAC 27, 35 considered different outcomes (RFS and CS), making the data unsuitable for a sub-analysis to detect any differences, if present, between EAC and SCC. Despite these limitations, we provided a comprehensive analysis of the association between CD34, CD133, Nucleostemin, OCT-4, NANOG and CD90 stemness markers and OS of patients affected by esophageal cancer. In summary, our meta-analysis revealed that high expression of CD34, CD133, and Nucleostemin was significantly associated with poor OS, suggesting that these stemness markers are promising prognostic factors in patients affected by esophageal cancer 60.

## Figures and Tables

**Table 1:** ** Characteristics of included studies.** SCC: squamous cell carcinoma; EAC: esophageal adenocarcinoma; IHC: immunohistochemistry; ISH: *in situ* hybridization; RFS: relapse-free survival; DSF: disease-free survival; OS: overall survival; CSS cause-specific survival; ECSS: esophageal cancer-specific survival

AUTHOR	YEAR	STEM CELL MARKER	COUNTRY	ETHNICITY	TUMOR TYPE		DETECTION METHOD	CUT-OFF VALUE	OUTCOME	HAZARD RATIO (REPORTED/ ESTIMATED)	NOS SCORE
					SCC	EAC					
Perry et al.	2015	CD34	UK	Caucasian	0	43	IHC	>0	RFS	E	6
Lu et al.	2015	CD133	China	Asian	154	0	IHC	Score ≥2 (0-3)	DFS OS	R	5
Shimada et al.	2012	NANOG and OCT-4	Japan	Asian	81	0	IHC	NANOG: Score >2 (0-5); OCT-4 Score >3 (0-5)	CSS	E	5
Zhang et al.	2010	Nucleostemin	China	Asian	62	0	ISH	>0	Lymphatic Metastasis	E	6
He et al.	2012	OCT-4	China	Asian	153	0	IHC	Score >3 (0-9)	OS	R	6
Okamoto et al.	2016	CD133	Japan	Asian	47	0	IHC	>80%	ECSS	R	6
Yang et al.	2017	OCT-4	Korea	Asian	127	0	IHC	Score ≥2 (0-3)	DFS OS	R	6
Nakajima et al.	2012	Nucleostemin and CD133	Japan	Asian	51	0	IHC	Nucleostemin >80%; CD133 >0	OS	R	7
Li et al.	2012	OCT-4	China	Asian	50	0	IHC	Score ≥3 (0-4)	OS	R	6
Goscinski et al.	2013	CD34	Norway	Caucasian	24	28	IHC	>25%	CS	E	5
Elpek et al.	2001	CD34	Turkey	Caucasian	53	0	IHC	≥92	OS	E	6
Igarashi et al.	1998	CD34	Japan	Asian	83	0	IHC	>116	OS	E	6
Hang et al.	2012	CD133	China	Asian	110	0	IHC	>1%	OS	E	7
Hwang et al.	2014	NANOG	Taiwan	Asian	41	0	IHC	>0	CS	E	5
Okamoto et al.	2013	CD133	Japan	Asian	86	0	IHC	>1%	OS	R	7
Kitadai et al.	1998	CD34	Japan	Asian	71	0	IHC	>43	OS	E	5
Sun et al.	2015	NANOG	China	Asian	149	0	IHC	>8 (0-12)	CS	E	6
Tanigawa et al.	1997	CD34	Japan	Asian	43	0	IHC	≥145	OS	R	6
Sarbia et al.	1996	CD34	Germany	Caucasian	130	0	IHC	>63	Lymphatic Metastasis	E	6

**Table 2:** Characteristics of antibodies used for staining procedures in the included studies. NA: not available

AUTHOR	YEAR	STEM CELL MARKER	ANTIBODY	DILUTION
Perry et al.	2015	CD34	Anti-CD34 (MCAP547, Serotec, Oxford, UK)	1:500
Lu et al.	2015	CD133	Anti-CD133 (Cloud-Clone Corp, Houston, Tex)	NA
Shimada et al.	2012	NANOG and OCT-4	Anti-NANOG (IHC-00205; Bethyl Laboratories, Montogomery, TX, USA) Anti-OCT4 (ab19857; Abcam)	NA
Zhang et al.	2009	Nucleostemin	Anti-human nucleostemin (R&D Systems, Minneapolis, MN, USA)	NA
He et al.	2012	OCT-4	Anti-OCT4 (AF1759, R&D Systems, Minneapolis, MN, USA)	1:40
Okamoto et al.	2016	CD133	Anti-CD133 (PROM-1, Abnova Corporation, Taipei, Taiwan)	1:250
Yang et al.	2017	OCT-4	Anti-Oct4 (Millipore, USA)	1:100
Nakajima et al.	2012	Nucleostemin and CD133	Anti-nucleostemin (A300-600A; Bethyl Laboratories, Montgomery, TX, USA) Anti-CD133 (130-092-395; Miltenyi Biotec, Auburn, CA, USA)	1:100 1:250
Li et al.	2012	OCT-4	Anti-human OCT4 (Santa Cruz Biotechnology, Inc. Santa Cruz, CA, USA)	NA
Goscinski et al.	2013	CD34	Anti-CD34 (QBend-10, Monosan, The Netherlands)	1:1000
Elpek et al.	2001	CD34	Anti-CD34 (Qbend-10, Dako, Glostrup, Denmark)	1:50
Igarashi et al.	1998	CD34	Anti-CD34 (Biogenesis Inc., Poole, UK)	1:500
Hang et al.	2012	CD133	Anti-CD133 (C24B9, Cell Signaling Technologies, Danvers, MA, USA)	1:100
Hwang et al.	2014	NANOG	Anti-NANOG (Epitomics, Burlingame CA, USA)	NA
Okamoto et al.	2013	CD133	Anti-CD133 (AC133, Miltenyi Biotec, Auburn, CA, USA)	1:10
Kitadai et al.	1998	CD34	Anti-CD34 (Nichirei, Tokyo, Japan)	1:300
Sun et al.	2015	NANOG	Anti-NANOG (Cell Signaling Technologies, Danvers, MA, USA)	1:200
Tanigawa et al.	1997	CD34	Anti-CD34 (QBend-10, Novocastra Labo, Newcastle, UK)	1:25
Sarbia et al.	1996	CD34	Anti-CD34 (QBend-10, Serotec, Oxford, UK)	NA

**Table 3:** ** Characteristics of patients enrolled in the included studies.** EGJ: esophagogastric junction; NA: not available; CT: chemotherapy; RT: radiotherapy.

AUTHOR	SAMPLE SIZE		SEX		AGE	FOLLOW UP (MONTHS)	TUMOR STAGE				TUMOR LOCATION			NEOADJUVANT THERAPY	
	Total Patients	Actual Patients	M	F			I	II	III	IV	EGJ/ lower	thoracic/middle	cervical/upper	CT	RT
Perry et al.	43	43	32	11	68,2	43 (3-122)	NA	NA	NA	NA	23	20		0	0
Lu et al.	154	154	119	35	≤60=76; >60=78	108	12	83	59	0	NA	NA	NA	0	0
Shimada et al.	114	81	72	9	64,5	40	11	38	36	8	NA	NA	NA	0	0
Zhang et al.	62	62	36	26	<60=29; >60=33	not mentioned	7	28	26	1	NA	NA	NA	0	0
He et al.	153	153	93	60	56,4	124 (118-155)	5	39	100	9	35	101	14	0	0
Okamoto et al.	47	47	40	7		42 (6-82)	12		35		14	27	6	47	0
Yang et al.	147	127	120	7	<65=34; >65=93	120	15	112			NA	NA	NA	0	0
Nakajima et al.	54	51	47	7	62 (36-74)	until relapse	2	11	33	8	NA	NA	NA	51	0
Li et al.	50	50	37	13	62 (47-72)	80	14	31	5		NA	NA	NA	0	0
Goscinski et al.	52	52	42	10	38-87	60	NA	NA	NA	NA	NA	NA	NA	0	0
Elpek et al.	53	53	30	23	42 (32-55)	24 (6-60)	26		27		22	19	12	0	0
Igarashi et al.	93	83	84	9	64,3 (44-83)	28-36	44		59		33	47	13	0	0
Hang et al.	110	110	61	49	57 (38-81)	24	11	65	34		14	72	24	0	0
Hwang et al.	41	41	39	2	54 (37-78)	13 (0,3-57,4)		7	23	11	NA	NA	NA	41	41
Okamoto et al.	86	86	73	13	64 (37-81)	until death	20	28	3	5	41	35	10	0	0
Kitadai et al.	119	71	107	12	63,5 (39-86)	120	67		52		NA	NA	NA	81	81
Sun et al.	149	149	112	37	54,0	until death	4	82	54	9	NA	NA	NA	0	0
Tanigawa et al.	43	43	28	15	65 (46-81)	24	18	9	6	10	15	20	8	2	6
Sarbia et al.	150	130	121	29	58 (35-82)	24-192	72		78		NA	NA	NA	0	0
